# Supplementary material for: Signature of spin-triplet exciton condensations in LaCoO3 at ultrahigh magnetic fields up to 600 T
Source: Nat Commun. 2023 Apr 4;14:1744. doi: 10.1038/s41467-023-37125-4 (PMC10073196; doi:10.1038/s41467-023-37125-4)
Supplement: Supplementary file 1 — Supplementary Information [file 41467_2023_37125_MOESM1_ESM.pdf]

Supplementary Information

Akihiko Ikeda,[1,2] Yasuhiro H. Matsuda,[1] Keisuke Sato,[3] Yuto Ishii,[1]
Hironobu Sawabe,[1] Daisuke Nakamura,[1] Shojiro Takeyama,[1] and Joji Nasu[4,5]

[1]*Institute for Solid State Physics, University of Tokyo, Kashiwa, Chiba 277-8581, Japan*

[2]*Department of Engineering Science,*
University of Electro-Communications, Chofu, Tokyo 182-8585, Japan

[3]*National Institute of Technology, Ibaraki College,*
Hitachinaka, Ibaraki 312-0011, Japan

[4]*Department of Physics, Tohoku University, Sendai, Miyagi 980-8578, Japan*

[5]*PRESTO, Japan Science and Technology Agency,*
Honcho Kawaguchi, Saitama 332-0012, Japan

(Dated: February 23, 2023)

I. SUPPLEMENTARY NOTE 1: EXPERIMENTAL DETAILS

A. Generation of 600 T using electromagnetic flux compression method in ISSP

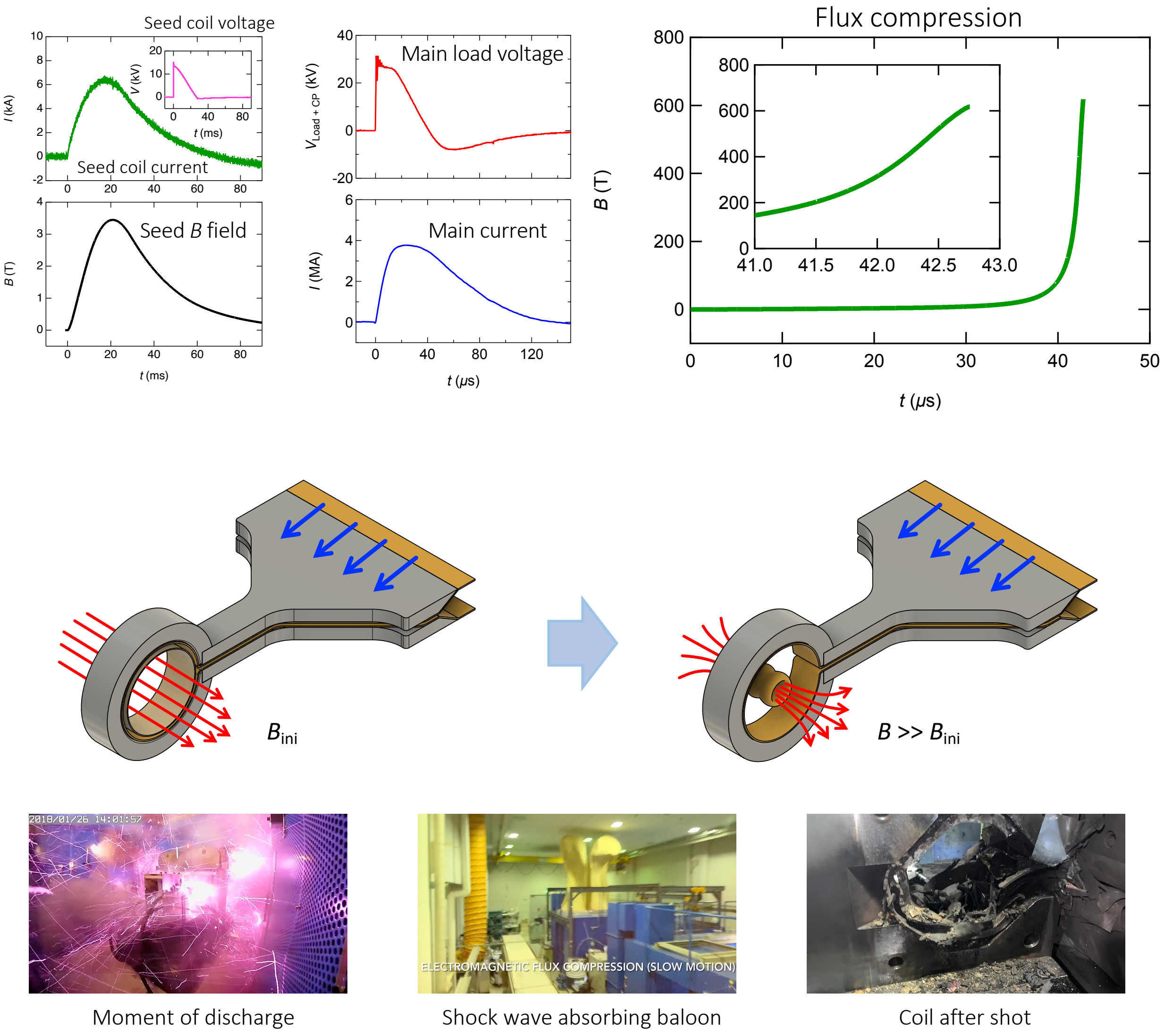

Supplementary Figure 1. The seed field generation, the main current used for the flux compression, and the typical magnetic field waveform in the present EMFC experiments.

The electromagnetic flux compression (EMFC) method is used to generate an ultrahigh magnetic field of 600 T in ISSP, University of Tokyo, that we implemented recently [1]. In the EMFC method, the ultrahigh magnetic field is generated by concentrating the seed magnetic flux into a small cross-section and volume. A cylindrical metallic liner is rapidly compressed using electromagnetic force for the flux concentration. The electromagnetic force originates in the current repulsion between

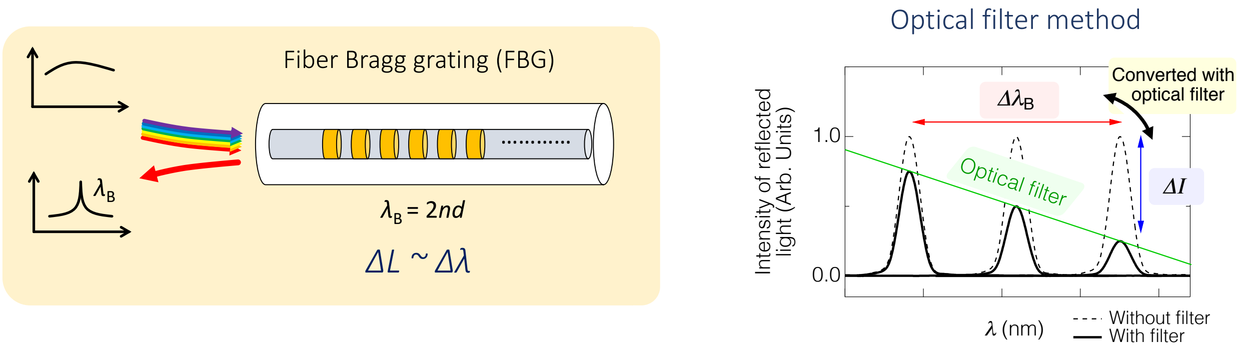

Supplementary Figure 2. Schematic drawing of the principle of FBG strain gauge and optical filter method for 100 MHz high-speed detection of strain.

the main coil and the induced current inside the metallic liner. A Helmholtz coil is used to generate a seed magnetic field of 3.4 T, where the energy stored in the sub capacitor bank is 0.95 MJ at a charging voltage of 13.8 kV. The Peak current of the main coil is 3.8 MA discharged with a charging voltage of 40 kV, where the energy stored in the main capacitor bank is 2.56 MJ. In Supplementary Figs. 1, the waveform of the seed magnetic field, main current and magnetic field are shown along with the schematic drawing of the flux compression and the pictures of the EMFC experiment in ISSP.

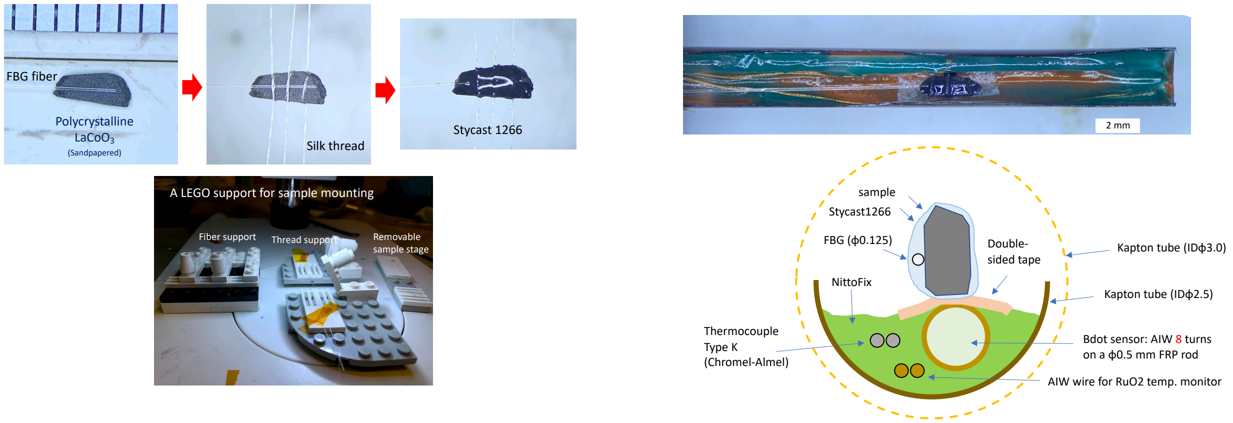

Supplementary Figure 3. Technique for gluing the sample and FBG fiber together using silk thread. A LEGO(R) support was used. The picture and the schematic cross-section of the sample probe are shown.

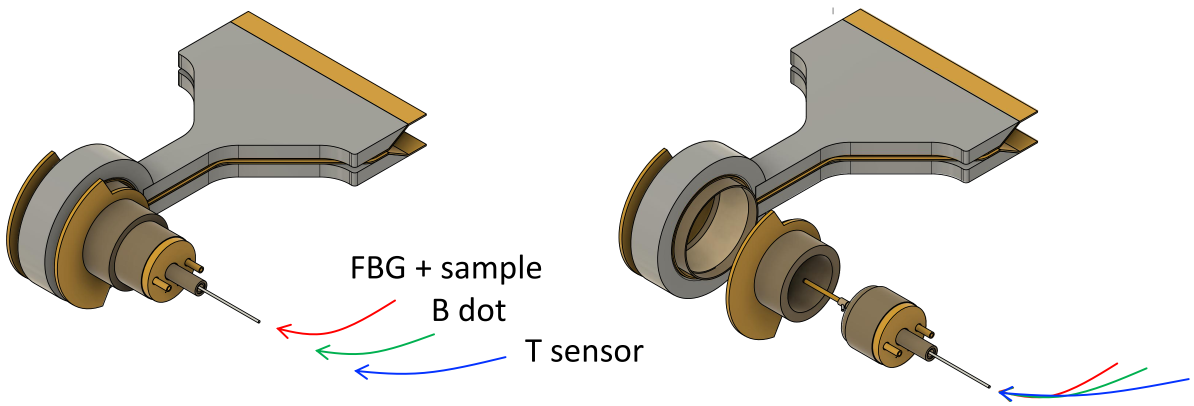

Supplementary Figure 4. The image of the vacuum chamber and the cryostat for the EMFC experiments. The sample and all the sensors are inserted from the right end of the image, being removable from the system.

B. High-speed magnetostriction measurement using fiber Bragg grating and optical filter method for the use up to 600 T

We have employed the original high-speed strain gauge utilizing the fiber Bragg grating (FBG) technique and optical filter method [2, 3] to measure the longitudinal magnetostriction of $LaCoO_3$ under ultrahigh magnetic fields generated using EMFC. Requirements for the magnetostriction measurement with EMFC are 100 MHz high speed and noiselessness. FBG is an optical fiber equipped with optical Bragg grating in the core, that we can use as an optical strain gauge as schematically shown in Supplementary Fig. 2. The strain of the optical fiber sensitively appears as the shift of the Bragg wavelength. Thus, the shift of the Bragg wavelength can be monitored optically from a distance, which is of benefit considering the large noise from the explosion and electric discharge involved in the EMFC technique. We used the optical filter method as the detection scheme [2]. This is because the required measurement speed is as high as 100 MHz in the EMFC experiment. In the optical filter method, the shift of the Bragg wavelength is converted to the change of the intensity of the optical signal as schematically shown in Supplementary Fig. 2. The change in the intensity of the optical signal is monitored by the InGaAs avalanche photodiode.

The strain of the sample is transmitted to the FBG fiber that is glued together [See Supplementary Fig. 3]. The gluing has been carried out in the following manner. First, two or three silk threads are used to tie the sample to the FBG fiber. This is performed on a base made from LEGO(R) bricks.

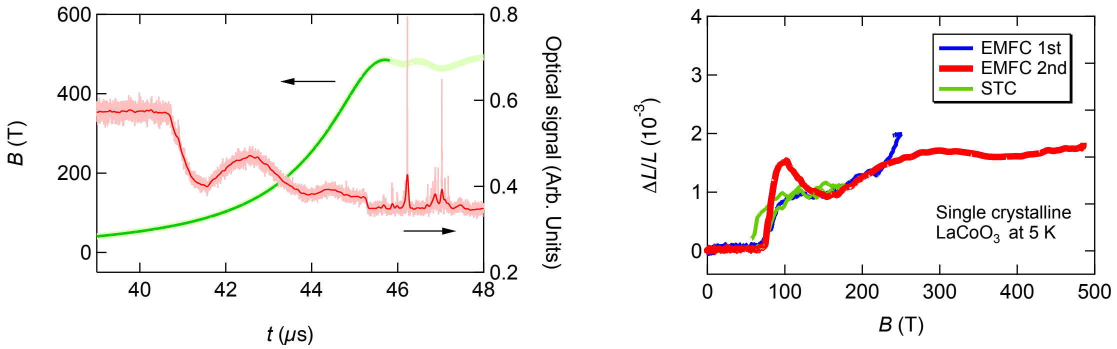

Supplementary Figure 5. Results of magnetostriction measurement using single crystalline $LaCoO_3$ up to 600 T at 5 K.

The bottom base is removed and STYCAST 1266 is used to glue the sample and the FBG fiber with the silk thread. The silk thread is used to make sure the FBG fiber is intact with the sample during the drying proccesse. The gluing method is the heart of the technique. The glued sample and FBG are placed on a Kapton sample holder tube. The induction type magnetic field sensor and temperature monitors of thermocouple or resistive RuO_2 tip are placed nearby in diameter of ϕ 2.5 mm [See Supplementary Fig. 3]. The probe equipped with the sample, FBG strain gauge, magnetic field sensor, and temperature sensors is inserted into a He flow type cryostat. The cryostat is placed at the field center inside a vacuum chamber made of bakelite parts, which serve as a thermal insulator and are also advantageous for the Cu liner implosion in the EMFC technique [See Supplementary Fig. 4].

C. Magnetostriction measurements with single crystalline samples

Single crystalline samples are measured up to 200 T as shown in Supplementary Fig. 5. It is difficult to observe the temperature dependence coherently with many pulses because single crystalline samples break themselves due to the field-induced phase transition taking place too rapidly. The strong vibrations of the data are present [See Supplementary Fig. 5]. The temperature-dependent data is disturbed and incomparable due to the re-gluing fresh samples to new optical fibers. On the other hand, polycrystalline samples are robust against the spin-state transitions triggered by the rapid magnetic field pulse. Poly-and single-crystalline samples show basically identical results with minor differences in the sharpness of the spin-state transitions.

D. Consideration on spin-orbit interactions

We comment on the difference between dV/V and dL/L. Spin-state originates purely in the electron spin ($\boldsymbol{S}$) when no spin-orbit interaction ($\boldsymbol{S} \cdot \boldsymbol{L}$) is in play. In that case, dL/L can represent dV/V. Actually, such a situation is common in transition metal oxides, where the orbital angular momentum ($\boldsymbol{L}$) is usually quenched. In the case of $LaCoO_3$, the orbital angular momentum is not completely quenched. Thus, an influence of spin-orbit interaction may result in $dV/V \neq dL/L$ in magnetostriction even for the polycrystalline sample used in the present study. In reality we may ignore this because it is reported small enough to be neglected in the present experiment by an electron resonance study showing the almost isotropic g-factors ($g_{\parallel} = 3.35$, $g_{\perp} = 3.55$) in $LaCoO_3$ [4].

We comment on another experimental fact indicating that we can ignore the spin-orbit couplings. Previously, we argued that the magnetization increases with increasing magnetic fields in β phase although magnetostriction is a constant, which may induce the spin-driven lattice change through spin-orbit coupling. We confirmed that this is not the case with measurements of magnetostriction and magnetization at room temperature, where only magnetization increases up to 50 T without any increase in magnetostriction [3]. It is reasonable to assume that the spin-orbit coupling, a local interaction, is temperature independent and is also absent at 78 and 108 K. This justifies our assumption that the longitudinal magnetostriction is proportional to lattice volume in the present study. The influence of orbital order is also unlikely either because we presently used a polycrystalline sample.

E. Effect of mechanical vibrations

We exclude the possibility of mechanical vibration as a source of the oscillating feature in 108 K data [see Fig. 3c in the main text] on the following basis. The key idea is temperature dependence. If the oscillating feature in 108 K data is actually a vibration, it should have appeared in the 78 K data with similar amplitude and frequency. We see only a small oscillating feature in the 78 K data above B_{c2}. The sharp slope features in 78 K are more apparent. 108 K is only 30 K above 78 K. The mechanical property is similar in this temperature change as reported in an ultrasound study [5]. Similarly, at the data 185 K, no negative slope is observed. This also indicates that the negative slopes of the data at 78 K and 108 K are intrinsic features.

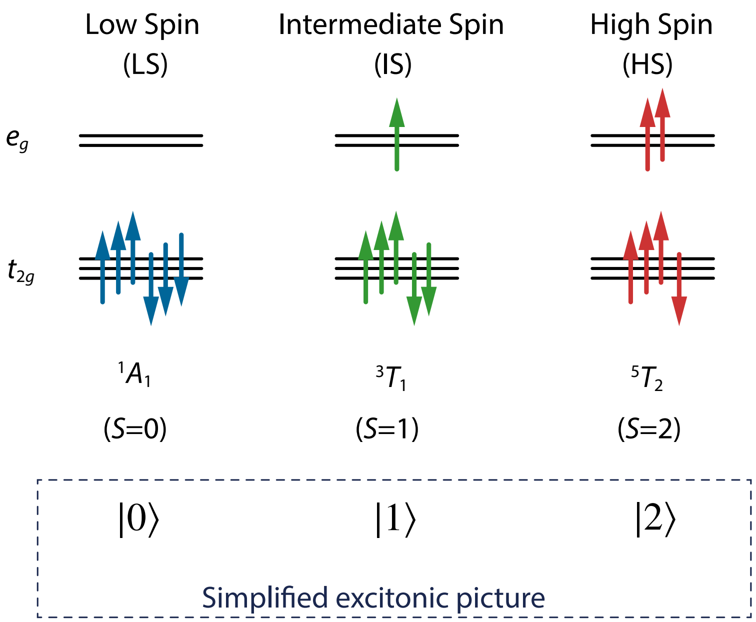

Supplementary Figure 6. Schematic picture of the three spin states and their excitonic representations simplified in the magnetic field.

Quantitatively, If the feature in 108 K is vibration, the shock propagation speed corresponds to 1.5 km/s with 250 kHz vibration and a sample size of 3 mm. In the same way, we obtain 3 km/s with 600 kHz vibration and a sample size of 3 mm. According to Ref. [5], the sound velocity change from 5 K to 108 K is 15 %. Thus the oscillating feature in 108 K data is too slow for a shock propagation vibration.

II. SUPPLEMENTARY NOTE 2: MEAN FIELD CALCULATION

We present the details of the theoretical model for the magnetic properties of trivalent cobalt ions ($3d^6$) in $LaCoO_3$ in an applied magnetic field and introduce the calculation method to address the model. We also show the results and discussion.

A. Model

We start from the five-orbital Hubbard model under the magnetic field to understand the properties of $3d$ electrons in cobalt ions. This model is composed of the following four terms: the transfer integrals between $3d$ orbitals in neighboring ions, $\mathcal{H}_t$, on-site Coulomb interactions

$\mathcal{H}_U$, crystalline electric field $\mathcal{H}_{\rm crys}$ with the octahedral symmetry, and Zeeman term $\mathcal{H}_Z$. Since there are five orbitals, which are occupied by six electrons per site, the Hamiltonian is extremely complicated. The competition between the local Hamiltonians, $\mathcal{H}_U$ and $\mathcal{H}_{\rm crys}$, is understood from the well-known Tanabe-Sugano diagram. In the d^6 configurations, three types of spin states, low-spin (LS), intermediate spin (IS), and high-spin states (HS) shown in Supplementary Fig. 6 play a crucial role in the magnetic properties of a local cobalt site. When the on-site Coulomb interactions are only taken into account, the ground state of the local d^6 configurations is the LS multiplet ^5D ($S = 2, L = 2$) corresponding to the HS state while the IS and LS are excited states. By introducing the crystalline electric field of the octahedral ligand arrangement, the ground state changes to the LS state where the t_{2g} orbitals are fully occupied. The IS can be a first excited state but does not become a ground state within a single ion system. Nevertheless, intensive experimental and theoretical studies on cobalt oxides suggested the possibility of the emergence of the IS state due to the competition between itinerant and localized natures of electrons.

Next, we discuss the effect of the Zeeman term $\mathcal{H}_Z$. The strong magnetic field lifts spin and orbital degeneracies, which allows us to extract a state with the Zeeman energy minimized in each spin-state manifold. Such states in LS, IS, and HS manifold are referred to as $|0\rangle$, $|1\rangle$, and $|2\rangle$, respectively. Here, we neglect the degeneracy surviving under the magnetic field in each spin state for simplicity. We construct the low-energy effective Hamiltonian in the subspace composed of the direct products of the three local states $|0\rangle$, $|1\rangle$, and $|2\rangle$ in the strong correlation limit. The effective Hamiltonian is written as

$$\mathcal{H} = \mathcal{H}_{\rm loc} + \mathcal{H}_{\rm int} + \mathcal{H}_{\rm trans} + \mathcal{H}_{\rm dual}. \tag{1}$$

The first term $\mathcal{H}_{\rm loc}$ represents the local energy levels of the IS and HS states, which is given by

$$\mathcal{H}_{\rm loc} = (E_1 - g_1 h) \sum_i n_i^{(1)} + (E_2 - g_2 h) \sum_i n_i^{(2)}, \tag{2}$$

where E_1 and E_2 are the on-site energies of $|1\rangle$ and $|2\rangle$ measured from the LS one, respectively, and $n_i^{(1)} = |1\rangle_i \langle 1|_i$ and $n_i^{(2)} = |2\rangle_i \langle 2|_i$ are the number operators at site i. The g factors for the IS and HS states are written as $g_1 = 2$ and $g_2 = 4$ under the magnetic field h. E_1 and E_2 should be positive because the ground state without magnetic fields is LS state.

The other terms in Eq. (1) are deduced from the second-order perturbation process with respect to $\mathcal{H}_t$. For simplicity, we neglect the orbital anisotropy related to bond-dependent transfer integrals in $\mathcal{H}_t$. First, we consider the diagonal part, where a local spin state is not changed by the perturbation

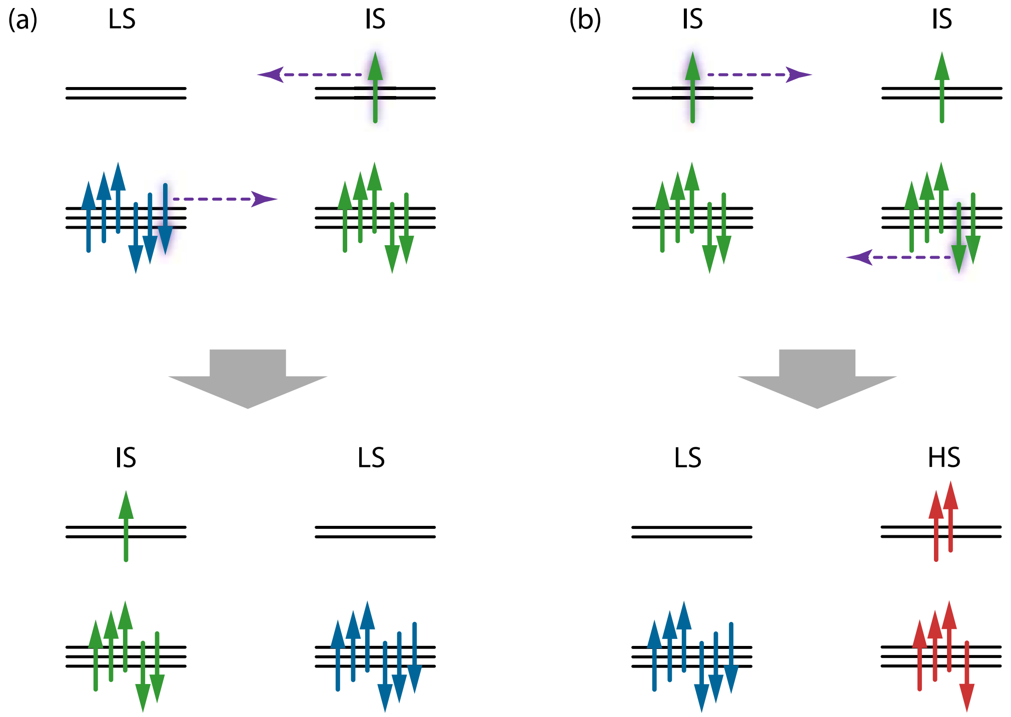

Supplementary Figure 7. Two types of second-order perturbation processes changing neighboring local spin states: (a) the exchange process between LS and IS states and (b) the process where two IS states are changed to LS and HS states, which is regarded as a fusion process of two single-excitons to a bi-exciton.

process. The transfer integrals between the e_g orbitals at neighboring sites is much larger than those involving the t_{2g} orbitals when the neighboring octahedra share their corners. In this case, the energy gain is yielded by the perturbation process for the IS-IS, LS-HS, LS-IS, and IS-HS configurations at neighboring sites. While the IS state does not become the ground state in a single ion system, one expects more perturbation processes involving the IS state than the HS because of the orbital degeneracy of the e_g orbitals in the IS state. Thus, we extract the IS-IS and LS-HS configurations as dominant contributions, and the corresponding Hamiltonian is given by

$$\mathcal{H}_{\mathrm{int}} = -J_{11} \sum_{\langle ij \rangle} n_i^{(1)} n_j^{(1)} - J_{02} \sum_{\langle ij \rangle} \left(n_i^{(2)} n_j^{(0)} + n_i^{(0)} n_j^{(2)} \right), \tag{3}$$

where $n_i^{(0)}$ is the number operator for the LS state. Note that J_{11} and J_{02} should be positive because of the second-order perturbation.

Next, we consider the exchange terms in the perturbation expansion. As suggested in Ref. 6, there are two dominant processes: the exchange process between neighboring LS and IS states and the change from the neighboring two IS states into LS and HS states. The former is interpreted

as the hopping of a single exciton to a neighboring site, $|0,1\rangle_{ij} \rightleftharpoons |1,0\rangle_{ij}$, and the latter as the fusion of two single excitons into a bi-exciton (and the fission of it), $|1,1\rangle_{ij} \rightleftharpoons |0,2\rangle_{ij}$. These contributions are given by

$$\mathcal{H}_{\rm trans} = -t \sum_{\langle ij \rangle} \left(|0,1\rangle_{ij} \langle 1,0|_{ij} + |1,0\rangle_{ij} \langle 0,1|_{ij} \right), \tag{4}$$

$$\mathcal{H}_{\rm dual} = -V \sum_{\langle ij \rangle} \left[|1,1\rangle_{ij} \left(\langle 2,0|_{ij} + \langle 0,2|_{ij} \right) + \left(|2,0\rangle_{ij} + |0,2\rangle_{ij} \right) \langle 1,1|_{ij} \right], \tag{5}$$

where $|k,k'\rangle_{ij} = |k\rangle_i \otimes |k'\rangle_j$.

The exchange constants, E_1, E_2, J_{11}, J_{02}, t, and V should be obtained from the perturbation procedure, but determining their values is a significantly tough problem. To avoid this, we regard these constants as external parameters. The signs of t and V cannot be determined directly. Fortunately, these can be inverted by an appropriate gauge transformation in the case of the bipartite lattice. Here, we consider the three-dimensional cubic lattice with the coordination number $z = 6$. In this case, the obtained phase diagram does not depend on the signs. Thus, we assume that t and V are positive hereafter.

B. Mean-field theory

Here, we show the details of the mean-field theory applied to the model Hamiltonian given in Eq. (1). We introduce two types of pseudospins, $\boldsymbol{\tau}_i$ and $\boldsymbol{\rho}_i$ at site i, which are defined by

$$\tau_i^x = |0\rangle_i \langle 1|_i + \text{H.c.}, \quad \tau_i^y = i\,|0\rangle_i \langle 1|_i + \text{H.c.}, \tag{6}$$

$$\rho_i^x = |1\rangle_i \langle 2|_i + \text{H.c.}, \quad \rho_i^y = i\,|1\rangle_i \langle 2|_i + \text{H.c.}, \tag{7}$$

respectively. Using these pseudospins, $\mathcal{H}_{\rm trans}$ and $\mathcal{H}_{\rm dual}$ are rewritten as

$$\mathcal{H}_{\rm trans} = -\frac{t}{2} \sum_{\langle ij \rangle} \left(\tau_i^x \tau_j^x + \tau_i^y \tau_j^y \right), \tag{8}$$

$$\mathcal{H}_{\rm dual} = -\frac{V}{2} \sum_{\langle ij \rangle} \left(\tau_i^x \rho_j^x + \rho_i^x \tau_j^x + \tau_i^y \rho_j^y + \rho_i^y \tau_j^y \right). \tag{9}$$

We apply the mean-field decoupling to $\mathcal{H}_{\rm int}$, $\mathcal{H}_{\rm trans}$, and $\mathcal{H}_{\rm dual}$, where two-sublattice orders are assumed. The order parameters are given by $\langle \tau^x \rangle_X$, $\langle \tau^y \rangle_X$, $\langle \rho^x \rangle_X$, $\langle \rho^y \rangle_X$, and $\langle n^k \rangle_X$, where $k = 0,1,2$ and $X (= A, B)$ is the sublattice index. The operator $\boldsymbol{\tau}$ corresponds to a mixing between the LS and IS states, and a nonzero $\langle \boldsymbol{\tau} \rangle_X$ suggests excitonic condensation, while a nonzero

expectation value of the operator $\boldsymbol{\rho}$ indicates a spontaneous hybridization between IS and HS states. Note that the U(1) symmetry appears to be present in $\mathcal{H}_{\rm trans}$ in the pseudospin space but it is due to the simplification of the effective model. If we carry out a more rigorous derivation of the effective model from the multi-orbital Hubbard model, this symmetry should be absent in the obtained Hamiltonian [7].

C. Mean-field result

We show the results of mean-field calculations for the effective model given in Eq. (1). Figure 8 shows the magnetic-field dependence of the mean-field energy, classical energies given by

$$\begin{cases} E_0 = 0, \\ E_{11} = E_1 - g_1 h - zJ_{11}/2, \\ E_{22} = E_2 - g_2 h, \\ E_{02} = (E_2 - g_2 h - zJ_{02})/2, \end{cases} \tag{10}$$

and the order parameters given by

$$\begin{cases} \bar{n}_k^{\pm} = \left|\langle n^{(k)}\rangle_A \pm \langle n^{(k)}\rangle_B\right|, \qquad (k=0,1,2) \\ \bar{\tau}^{\pm} = \sqrt{(\langle\tau^x\rangle_A \pm \langle\tau^x\rangle_B)^2 + (\langle\tau^y\rangle_A \pm \langle\tau^y\rangle_B)^2}, \\ \bar{\rho}^{\pm} = \sqrt{(\langle\rho^x\rangle_A \pm \langle\rho^x\rangle_B)^2 + (\langle\rho^y\rangle_A \pm \langle\rho^y\rangle_B)^2}. \end{cases} \tag{11}$$

We choose the parameters in Eq. (1) such that the LS is realized in the ground state in the absence of the magnetic field and this state changes to the LS-HS order and uniform HS state successively by applying the magnetic field in the classical case where $\mathcal{H}_{\rm trans}$ and $\mathcal{H}_{\rm dual}$ are dropped. The LS-HS order is regarded as the exciton solid (ES). The previous experimental studies suggest the situation [3, 8, 9], and thereby, we focus on the parameters shown in Supplementary Fig. 8, which satisfy the above conditions.

The parameters in Supplementary Fig. 8(b) are the same as those of the mean-field result shown in the main text. In this case, we find two excitonic phases, EC1 and EC2, in addition to the uniform LS and HS phases. The EC1 state is characterized by the uniform excitonic order parameters $\bar{\tau}^+$ and $\bar{\rho}^+$. Since $\bar{n}_2$ is smaller than $\bar{n}_0$ and $\bar{n}_1$, this state is interpreted as condensation of single-excitons $|1\rangle$. On the other hand, in the EC2 state, the staggered component of the order parameters continuously appears from the EC1 phase, indicating the second-order phase transition between EC1 and EC2.

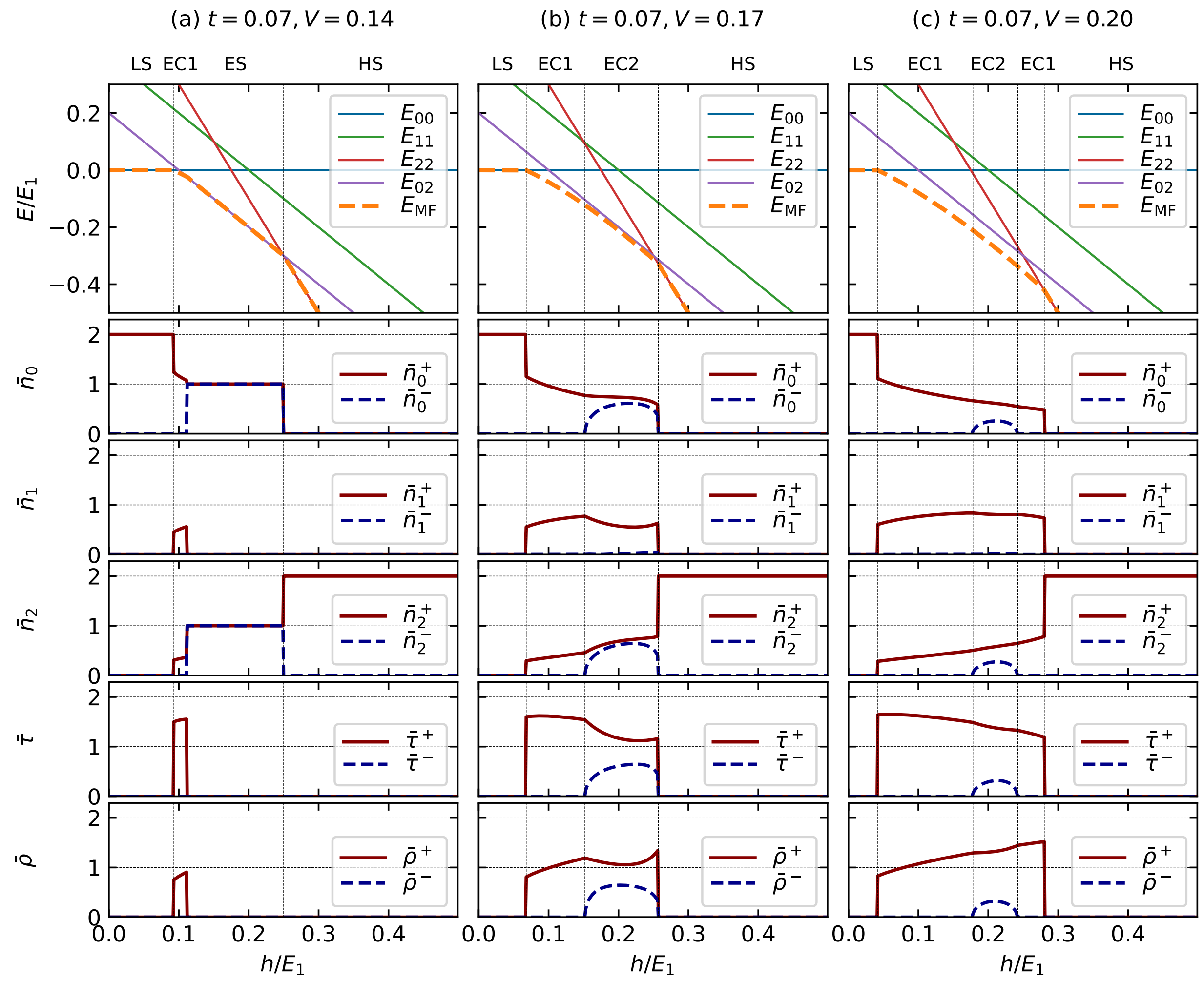

Supplementary Figure 8. Mean-field results for the effective model given in Eq. (1) at (a) $V = 0.14$, (b) $V = 0.17$, and (c) $V = 0.20$. The other parameters are chosen to be $(E_1, E_2, J_{11}, J_{02}, t) = (1.0, 0.7, 0.2, 0.05, 0.07)$. The top panels represent the mean-field energy and classical energies given in Eq. (10) as functions of h. The others show the field dependence of the mean-fields given in Eq. (11). The energy unit in the present calculations is E_1, which is estimated as $\sim$ 10 meV correspinding to $\sim$ 100 T.

Among them, $\bar{n}_1^-$ is much smaller than the others, which suggests that the solidification of bi-excitons emerges together with excitonic condensation. Thus, the EC2 is regarded as an excitonic supersolid. This phase is shrunk with increasing V as shown in Supplementary Fig. 8(c). On the other hand, Supplementary Fig. 8(a) indicates that the decrease of V changes the EC2 to the classical ES state. In this case, the phase transition between EC1 and ES is of the first order. Therefore, we deduce that the fusion of the two single-excitons, caused by $\mathcal{H}_{\rm dual}$, plays a crucial

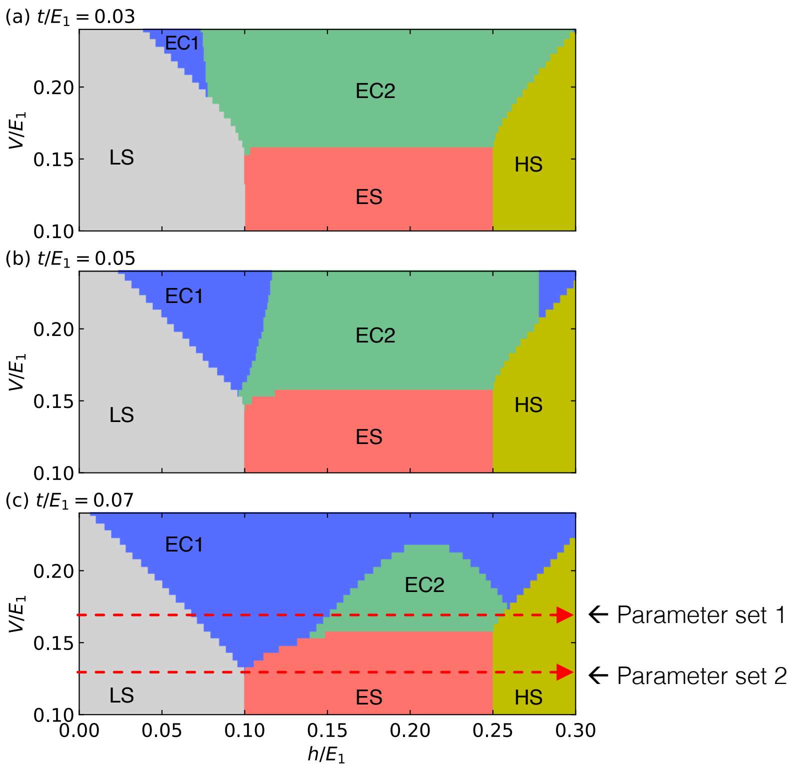

Supplementary Figure 9. Phase diagram on the parameter space of V/E_1 and h/E_1 with the parameter variations of t = (a) 0.03, (b) 0.05, and (c) 0.07. The other parameters are chosen to be $(E_1, E_2, J_{11}, J_{02})$ = (1.0, 0.7, 0.2, 0.05). The horizontal dashed arrows indicate the parameter sets introduced in the main text.

role in inducing the EC2 state and the second-order transition to the EC1.

The phase diagrams on the plane of V/E_1 and h/E_1 are shown in Supplementary Figs. 9(a)-9(c) with the variations of t/E_1 = 0.03, 0.05, and 0.07, respectively. The other parameters are chosen to be $(E_1, E_2, J_{11}, J_{02})$ = (1.0, 0.7, 0.2, 0.05). The phase diagrams indicate that the competition is in play between the classical interaction terms $\mathcal{H}_{\text{int}}$ and the quantum terms $\mathcal{H}_{\text{trans}}$, $\mathcal{H}_{\text{dual}}$. And also the competition is in play between the quantum terms $\mathcal{H}_{\text{trans}}$ and $\mathcal{H}_{\text{dual}}$. They show that the EC1, EC2, and ES phases emerge between the LS and HS phases. With increasing V/E_1, the quantum phases of EC1 and EC2 emerge with suppression of the classical solid ES. With increasing t/E_1, EC1 becomes more stable as compared to EC2. With t/E_1 = 0.07 and V/E_1 = 0.17, the transition from LS to EC1 and then to EC2 appears, which is the parameter set 1 presented in the main text and Supplementary Fig. 9(c). With t/E_1 = 0.07 and V/E_1 = 0.13, the transition from LS to ES and then to HS appears, which is the parameter set 2 presented in the main text and Supplementary

Fig. 9(c).

D. Relation to the experimental results

We argue that the experimentally observed transitions from LS $\leftrightarrow \beta \leftrightarrow \gamma$ possibly correspond to the successive phase transitions in the calculated results, LS $\leftrightarrow$ EC1 $\leftrightarrow$ EC2, considering the experimentally observed features that two phases appear in high magnetic fields, which show continuous change of exciton density as a function of magnetic fields as shown in Supplementary Fig. 3g. We also argue that the experimentally observed α phase originates in the ES phase in Supplementary Fig. 8a, considering the wide plateau of exciton density in ES and the α phase.

We tentatively compare the transition fields of the experiment and the calculation. E_1, the energy of isolated IS state, is reported to be ~ 0.2 eV with a CoO_6 cluster calculation in Ref. [10], which is assumed to be unity in the calculation. Then, we obtain $h/E_1 = 0.1 \rightarrow 172.7$ T as a scale of the horizontal axis of Supplementary Figs. 8a-8c, with the Zeeman energy of $E = g\mu_{\mathrm{B}}BS_z$. We obtain the transition fields of (161 T, 193 T, 432 T), (117 T, 263 T, 444 T), and (73 T, 307 T, 418 T, 485 T) for Supplementary Figs. 8a-8c. As an order estimation, the obtained transition fields well account for the experimental result.

The negative slope in the β phase may result from the non-trivial spin-lattice coupling found in a high-resolution x-ray diffraction study, where the lattice volume of $|1\rangle$ is claimed to be smaller than a pair of neighboring site $|0\rangle$ and $|2\rangle$ [11]. Taking this into account, increasing the weight of $|1,1\rangle$ will contract the lattice volume in EC1 with increasing magnetic fields. On the other hand, increasing the weight of $|0,2\rangle$ state will expand the lattice volume in EC2.

We note that the α phase may emerge with modified interaction parameters as compared to those for the β and γ phases, as ES appears with the variation of the HS-IS duality parameter V suppressing the appearance of EC1 and EC2. The modification of the parameter should occur due to the strong lattice contraction, which enhances the localization nature of excitons, resulting in exciton superlattice formation in the α phase. Such lattice contraction in the course of the β-α phase transition above 100 T is actually observed in Ref. [3]. Further microscopic understanding of the lattice change affecting the interaction parameters will be an interesting work in the future which will be accomplished using the state-of-the-art technique utilizing an x-ray free electron laser and a portable generator of ultrahigh magnetic fields [12].

SUPPLEMENTARY REFERENCES

[1] D. Nakamura, A. Ikeda, H. Sawabe, Y. H. Matsuda, and S. Takeyama, Rev. Sci. Instrum. **89**, 095106 (2018).

[2] A. Ikeda, T. Nomura, Y. H. Matsuda, S. Tani, Y. Kobayashi, H. Watanabe, and K. Sato, Rev. Sci. Instrum. **88**, 083906 (2017).

[3] A. Ikeda, Y. H. Matsuda, and K. Sato, Phys. Rev. Lett. **125**, 177202 (2020).

[4] S. Noguchi, S. Kawamata, K. Okuda, H. Nojiri, and M. Motokawa, Phys. Rev. B **66**, 094404 (2002).

[5] T. Sin Naing, T. Kobayashi, Y. Kobayashi, M. Suzuki, and K. Asai, J. Phys. Soc. Jpn. **75**, 084601 (2006).

[6] A. Hariki, R.-P. Wang, A. Sotnikov, K. Tomiyasu, D. Betto, N. B. Brookes, Y. Uemura, M. Ghiasi, F. M. F. d. Groot, and J. Kuněs, Phys. Rev. B **101**, 245162 (2020).

[7] J. Nasu, T. Watanabe, M. Naka, and S. Ishihara, Phys. Rev. B **93**, 205136 (2016).

[8] M. M. Altarawneh, G. W. Chern, N. Harrison, C. D. Batista, A. Uchida, M. Jaime, D. G. Rickel, S. A. Crooker, C. H. Mielke, J. B. Betts, J. F. Mitchell, and M. J. R. Hoch, Phys. Rev. Lett. **109**, 037201 (2012).

[9] A. Ikeda, T. Nomura, Y. H. Matsuda, A. Matsuo, K. Kindo, and K. Sato, Phys. Rev. B **93**, 220401(R) (2016).

[10] M. W. Haverkort, Z. Hu, J. C. Cezar, T. Burnus, H. Hartmann, M. Reuther, C. Zobel, T. Lorenz, A. Tanaka, N. B. Brookes, H. H. Hsieh, H. J. Lin, C. T. Chen, and L. H. Tjeng, Phys. Rev. Lett. **97**, 176405 (2006).

[11] P. G. Radaelli and S. W. Cheong, Phys. Rev. B **66**, 094408 (2002).

[12] A. Ikeda, Y. H. Matsuda, X. Zhou, S. Peng, Y. Ishii, T. Yajima, Y. Kubota, I. Inoue, Y. Inubushi, K. Tono, and M. Yabashi, Appl. Phys. Lett. **120**, 142403 (2022).
